# Diagnostic ‘omics’ for active tuberculosis

**DOI:** 10.1186/s12916-016-0583-9

**Published:** 2016-03-23

**Authors:** Carolin T. Haas, Jennifer K. Roe, Gabriele Pollara, Meera Mehta, Mahdad Noursadeghi

**Affiliations:** Division of Infection and Immunity, University College London, Cruciform Building, Gower Street, London, WC1E 6BT UK

**Keywords:** Diagnostics, Disease, -Omics, Tuberculosis

## Abstract

The decision to treat active tuberculosis (TB) is dependent on microbiological tests for the organism or evidence of disease compatible with TB in people with a high demographic risk of exposure. The tuberculin skin test and peripheral blood interferon-γ release assays do not distinguish active TB from a cleared or latent infection. Microbiological culture of mycobacteria is slow. Moreover, the sensitivities of culture and microscopy for acid-fast bacilli and nucleic acid detection by PCR are often compromised by difficulty in obtaining samples from the site of disease. Consequently, we need sensitive and rapid tests for easily obtained clinical samples, which can be deployed to assess patients exposed to TB, discriminate TB from other infectious, inflammatory or autoimmune diseases, and to identify subclinical TB in HIV-1 infected patients prior to commencing antiretroviral therapy. We discuss the evaluation of peripheral blood transcriptomics, proteomics and metabolomics to develop the next generation of rapid diagnostics for active TB. We catalogue the studies published to date seeking to discriminate active TB from healthy volunteers, patients with latent infection and those with other diseases. We identify the limitations of these studies and the barriers to their adoption in clinical practice. In so doing, we aim to develop a framework to guide our approach to discovery and development of diagnostic biomarkers for active TB.

## Background

Making an early and definitive diagnosis of active tuberculosis (TB) infection is vital both at the individual and population level, thus reducing morbidity, mortality and transmission. The notoriously pleiotropic presentation of TB disease means that clinicians rely heavily on confirmatory diagnostics [1]. This review will assess how -omics based technology is poised to push this field beyond the limitations of currently available tests.

### Currently available *Mycobacterium tuberculosis* (Mtb) diagnostic approaches

The gold standard for microbiological diagnosis of Mtb relies on identification of the organism from clinical specimens. Microscopy, being rapid and affordable, remains the first-line diagnostic approach [2–4], but its sensitivity is both operator dependent and reliant on the abundance of Mtb in the sample [2]. Culture of Mtb improves sensitivity [5], but has inherent drawbacks – Mtb growth in vitro is fastidious and has a slow generation time (20–22 h) [6], and thus it takes weeks to identify Mtb from samples. Nevertheless, matrix-assisted laser desorption ionization-time of flight (MALDI-TOF) mass spectrometry and nucleic acid amplification tests (NAATs) may soon accelerate this step for the identification of positive cultures [7–11]. Liquid broth-based culture circumvents slow growth and subjective colony detection of Mtb on solid agar [12], improving both detection time and sensitivity compared to solid media cultures [4, 5, 13]. However, automated liquid culture systems necessitate significant laboratory infrastructure, and therefore other manual TB culture methods have been recommended for resource-limited settings [2]. Microscopic observation of drug sensitivity (MODS) uses inverted light microscopy to identify the typical cording pattern of Mtb in liquid culture; it is cost-effective in resource-limited settings and has similar or superior sensitivity and specificity to established culture systems [2, 14–16]. However, it still requires both skilled personnel and laboratory containment facilities, making it unsuitable for all settings and certainly not a point-of-care test.

An alternative approach to culture is Mtb antigen detection, best illustrated by the use of Mtb lipoarabinomannan in urine as a point-of-care diagnostic immunochromatographic assay. Although rapid and low cost, this test only achieves high sensitivity (>70 %) in HIV-TB co-infected patients with advanced immunodeficiency (CD4 < 200), limiting its diagnostic utility in an unselected population [17].

NAATs aim to detect Mtb directly from clinical specimens [7, 18], but only the line probe assay and Xpert MTB/RIF have been endorsed by the Word Health Organization for use in low- to middle-income countries [19, 20]. The line probe assay simultaneously detects Mtb and common rifampicin and isoniazid resistance mutations, but has a sensitivity of 58–80 % [21, 22] and still necessitates laboratory PCR facilities beyond the reach of many resource-limited settings. In contrast, the Xpert MTB/RIF platform performs PCR reactions within proprietary cartridges, making it a rapid diagnostic test [23]. Smear-positive sputum samples of confirmed pulmonary TB have 99 % sensitivity and a pooled specificity of 98 % [24]. Xpert MTB/RIF also detects the most common rifampicin resistance mutations in the Mtb *rpoB* gene, a proxy for multidrug-resistant TB, with a pooled sensitivity and specificity of 95 % and 98 %, respectively [20, 24]. However, Xpert MTB/RIF has a lower sensitivity in smear-negative sputum samples (68 %), and its sensitivity in extrapulmonary TB samples is highly variable (median 77.3 %, range 25.0–96.6 %) [24–27], leaving a significant proportion of TB disease reliant on sub-optimal accuracy from diagnostic tests.

### Whole genome pathogen sequencing

Recent advances in genomics offer the opportunity to advance TB diagnostics by improving bacterial detection. Whole genome sequencing (WGS) of clinical Mtb isolates has been used to retrospectively track Mtb transmission events [28, 29], discriminate between re-infection and relapse cases [30], and identify drug resistance-conferring mutations [31, 32]. Like NAATs, WGS provides both diagnostic confirmation of the presence of Mtb and information about antibiotic susceptibility using publicly available databases of annotated drug resistance and susceptibility mutations [33, 34]. WGS may yield results in a clinically relevant time frame, identifying the organism 1–3 days after a liquid culture flags positive [35, 36].

Excitingly, two recent studies propose faster diagnostic confirmation using WGS by successfully sequencing Mtb genomes directly from uncultured sputum samples [37, 38]. However, the ability to recover Mtb genome sequences also from smear- and culture-negative sputum samples (derived from previously diagnosed TB patients after anti-TB therapy) [38] emphasises that DNA-based techniques cannot discriminate between active disease and cleared infections, where DNA from dead mycobacteria may remain detectable.

### Host response-based diagnostics

In part owing to deficiencies in current diagnostics, around 42 % of notified cases are treated presumptively for TB disease [1]. In these circumstances, diagnostic confidence can be offered by the host response to Mtb infection: non-specific syndromic changes, such as anaemia, can be predictive of the likelihood of TB disease [39], and histopathological changes, such as caseating granulomata, support a diagnosis of tuberculosis [40] but are clearly limited by availability of diagnostic sampling of the site of disease. More dynamic pathological changes can now also be detected through imaging modalities such as CT-PET scanning [41], but these are still being evaluated and are not readily available. The host response to Mtb infection is also exploited in tuberculin skin tests (TSTs) and interferon gamma (IFN-γ) release assays (IGRAs): these are commonly used to diagnose asymptomatic ‘latent’ TB infection (LTBI; reviewed extensively elsewhere [42–44]), but they lack sensitivity or specificity in the diagnosis of active disease [45, 46]. Extensive research efforts have evaluated -omic technologies (Box 1) to screen host responses that might ultimately lead to better diagnostic tests for TB.

#### Blood transcriptomics

Over 20 studies examining the human transcriptional response to TB have been published since the first paper in 2007 [47] (Table 1). Despite this, no diagnostic test for TB utilising this technology exists. A number of reasons may account for this. Several of the studies were designed with the intention of exploring the immunopathogenesis of TB [48–53] rather than identifying diagnostic markers. Others have aimed at evaluating the treatment response to TB with a view to finding new surrogate markers of success for both clinical management and use in trials of new therapies [54, 55]. Of those designed to derive signatures that would discriminate active TB from health or other disease states, only a handful have a case definition of active TB based on microbiological confirmation, validation of their signatures in independent cohorts and evaluation of the diagnostic accuracy of the signature. We have focused on these studies in greater detail in this review.

**Table 1:** Transcriptomic studies

Study	Sample	Dataset GSE number	Country	Classes	Number	HIV status	Case definition					Independent test set^c^	Validation set^d^	Evaluation of accuracy	Signature size
							Prior TB treatment^a^	TB location	Microbiologically proven^b^	TST	IGRA				
Maertzdorf et al., 2015 [62]	WB	74092	India	TB	113	–	0	P	Y			Y	Y	Y	TB vs. LTB/HV 4
				LTBI	56					+/–	+/–				
				HC	20					–					
Walter et al., 2015 [61]	WB	73408	USA	TB	109	–		P	Y			Y	Y	Y	
				LTBI							+				
				Pneumonia							–				
Anderson et al., 2014 [60]	WB	39941	South Africa, Malawi, Kenya	CCTB	95	–		P, EP	Y			Y	Y	Y	TB vs. LTBI 42 TB vs. OD 51
				CNTB	27	–		P, EP	U						
				LTBI	68	–				+	+				
				OD	140	–					–				
				CCTB	51	+		P, EP	Y						
				CNTB	17	+		P, EP	U						
				LTBI	0	+				+	+				
				OD	93	+					–				
Cai et al., 2014 [58]	PBMC	54992	China	TB	173		0	P	Y			Y	N	Y	TB vs. HV 1, TB vs. LTBI 1
				LTBI	148						+				
				HC	51						–				
Dawany et al., 2014 [63]	PBMC	50834	South Africa	TB	21	+	Y	P	Y			N	Y	Y	HIV vs. HIV/TB 251
				HC	22	+									
Kaforou et al., 2013 [59]	WB	37250	South Africa, Malawi	TB	97	–	<1d	P, EP	Y			Y	Y	Y	TB vs. LTBI 27, TB vs. OD 44
				LTBI	83	–				+	+				
				OD	83	–					+/–				
				TB	97	+	<1d	P, EP	Y						
				LTBI	84	+				+	+				
				OD	92	+					+/–				
Bloom et al., 2013 [48]	WB	42834	UK & France	TB	35	–	0	P	Y			Y	Y	Y	TB vs. OD 144
				Sarcoid	61	–									
				Pneumonia	14	–									
				Lung cancer	16	–									
				HC	113	–					–				
Verhagen et al., 2013 [98]	WB	41055	Venezuela	TB	9	–	0	P		+	+	N	Y	Y	TB vs. LTBI 5
				LTBI	29	–				+	+				
				HC	25	–				–	–				
				Pneumonia	18	–									
Cliff et al., 2012 [54]	WB	3134836238	South Africa	TB	27	–	0, 1/4/26 w	P	Y			Y	Y	Y	Treatment 62
Maertzdorf et al., 2012 [51]	WB	34608	Germany	TB	8	–	0	P	U			N	N	Y	
				LTBI	4	–					+				
				HC	14	–					–				
				Sarcoid	18	–									
Ottenhof et al., 2012 [52]	PBMC	56153	Indonesia	TB	23	–	0, 8w, 28w	P	Y			N	N	N	
				HC	23										
Bloom et al., 2012 [55]	WB	40553	South Africa, UK	TB	37	–	0, 2w, 2 m, 6 m, 12 m	P	Y			Y	Y	N	TB vs. LTBI 664 treatment 320
				LTBI	38	–					+				
Lesho et al., 2011 [99]	PBMC	N/A	USA	TB	5	–		P	Y	+		N	N	Y	TB vs. LTBI vs. BCG vacc vs. HC 127
				LTBI	6	–				+					
				BCG vacc	5	–									
				HC	7	–				–					
Maertzdorf et al., 2011 [56]	WB	25534	South Africa	TB	33	–	0	P	Y			N	N	Y	TB vs. LTBI 5
				LTBI	34	–									
				HC	9	–									
Maertzdorf et al., 2011 [50]	WB	28623	The Gambia	TB	46	–	0	P	Y			N	N	N	
				LTBI	25	–				+					
				HC	37	–				0					
Lu et al., 2011 [100]	PBMC	27984	China	TB	46	–	<4w	P	Y			Y	Y	Y	TB vs. LTBI 3
				LTBI	59	–					+				
				HC	26	–					–				
Berry et al., 2010 [57]	WB	19491194441944319442194391943522098	UK, South Africa	PTB	54		0	P	Y			Y	Y	Y	TB vs. health 393 TB vs. OD 86
				LTBI	69					+	+				
				HC	24					–	–				
				OD	96										
Stern et al., 2009 [53]	PBMC	N/A	Colombia	TB	1			P	Y	+		N	N	N	
				LTBI	1					+					
				HC	1					–					
Jacobsen et al., 2007 [101]	PBMC	6112	Germany	TB	37	–	Y	P, EP	Y	+		N	Y	N	TB vs. LTBI vs. HC 3
				LTBI	22					+					
				HC	15					–					
Mistry et al., 2007 [47]	WB	N/A	South Africa	TB	10	–	0	Y				N	N	Y	TB vs. cured vs. LTBI vs. recurrent 9
				Cured TB	10	–									
				LTBI	10	–				+					
				Rec TB	10	–									

*WB* Whole blood, *PBMC* Peripheral blood mononuclear cells, *TB* Active tuberculosis, *LTBI* Latent TB infection, *HC* Healthy controls, *OD* Other diseases, *CCTB* Culture-confirmed TB, *CNTB* Culture-negative TB, *EP* Extrapulmonary, *P* Pulmonary, *Y* Yes, *N* No

^a^Number of days (d), weeks (w) or months (m) on treatment at time of sampling

^b^U if unclear whether all TB cases were microbiologically confirmed, e.g. if diagnosis was based on Mtb culture or chest X-ray or TB symptoms, or if microbiologically proven and unproven TB cases were grouped together

^c^Never involved in training the model

^d^New, independent set of samples

**Fig. 1:**
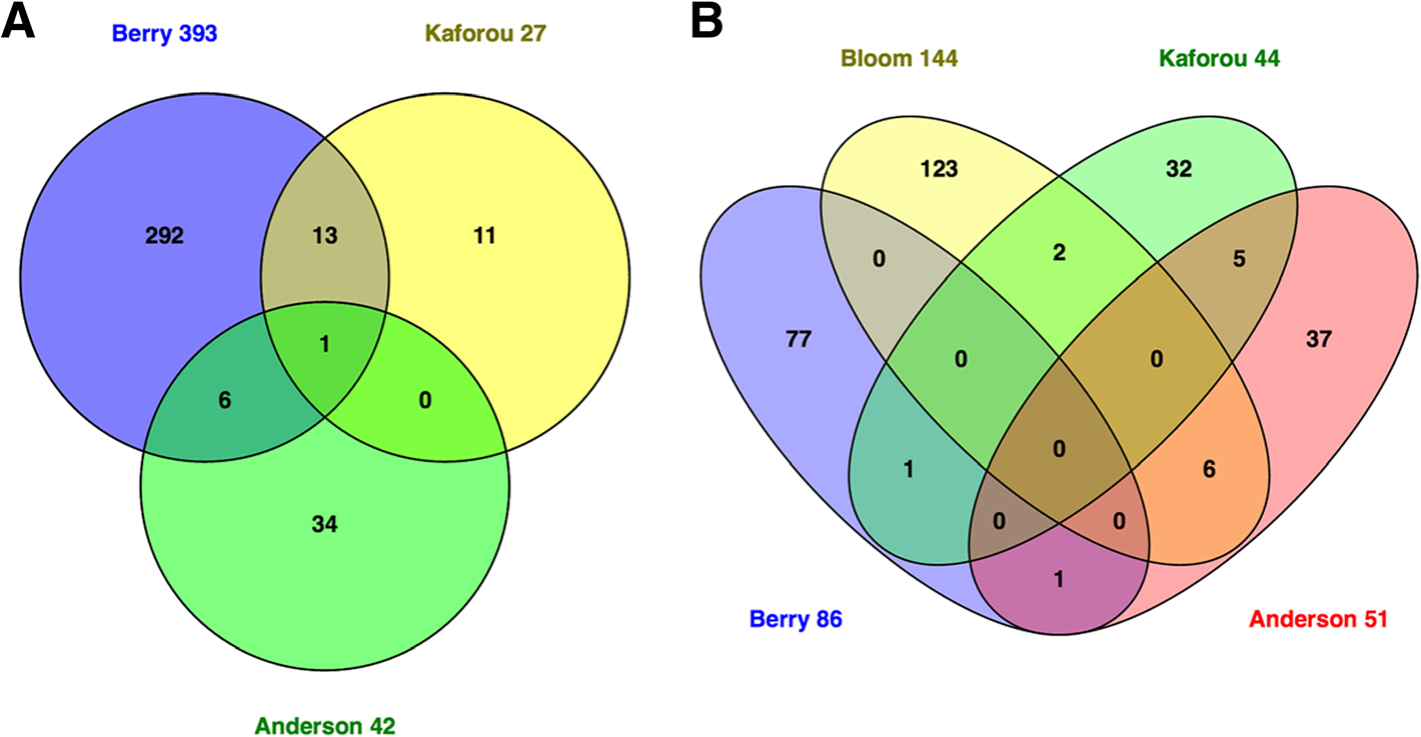
Venn diagrams of selected published transcriptomic signatures. Signatures were compared by gene symbol annotation, and the overlap visualised with Venn diagrams [115]. Since not all transcripts are annotated with a gene name, the gene numbers displayed in the Venn diagrams may not add up to the number of transcripts in the published signature. **a** Gene signatures that distinguish TB cases from healthy controls (including latently infected subjects). Berry 393 = 393-transcript signature of TB versus healthy states (LTBI and healthy controls) [57]; Kaforou 27 = 27-transcript signature of TB versus LTBI [59]; Anderson 42 = 42-transcript signature of TB versus LTBI [60]. **b** Gene signatures that distinguish TB cases from other diseases. Berry 86 = 86-transcript TB-specific signature [57]; Bloom 144 = 144-transcript signature of TB versus other pulmonary disease [55]; Kaforou 44 = 44-transcript signature of TB versus OD [59]; Anderson 51 = 51-transript signature of TB versus OD [60]. *TB* Tuberculosis, *LTB* Latent tuberculosis infection, *HC* Healthy controls, *OD* Other disease

Published transcriptional signatures for active TB vary in size and show surprisingly limited overlap between studies (Fig. 1). Nevertheless, common functional annotations associated with gene signatures of active TB have been observed in some studies. These include FCGR signalling [50, 56], interferon signalling [52, 57], and complement pathways [54, 58]. In addition to variations in study design, differences in patient demography, site and duration of TB disease, time on treatment and technical differences in the methodology of transcriptional profiling may have contributed to the diversity in signatures. The use of whole blood with and without globin depletion, or fractionated peripheral blood mononuclear cells for transcriptional profiling is likely to cause significant confounding. In addition, the use of different array platforms necessitates cross-comparison using a common feature such as gene name, but this may be insufficient because discriminating signatures in different studies may include unannotated probes with no gene name, or diverse probes for the same gene that do not give concordant signals.

Amongst the most highly cited studies, Berry et al. [57] described a 393-transcript signature of active TB versus healthy states, derived from a UK population (training set) and validated in a UK test set as well as in an independent South African cohort. Active TB cases were defined as culture-positive pulmonary TB with radiographic changes and whole blood transcriptomic samples were acquired prior to any TB treatment. Patients with LTBI were defined by the absence of signs or symptoms of active TB, and a positive IGRA and TST. Healthy controls had no symptoms or signs of TB and a negative IGRA and TST. Differentially expressed genes between active TB and healthy states (both LTBI and healthy controls) were identified in the training set using expression-level, statistical filters and hierarchical clustering. Machine learning and k-nearest neighbour class prediction showed a sensitivity and specificity of 61.67 and 83.75 %, respectively, in the UK test cohort, and of 94.12 and 96.67 %, respectively, in the South African validation cohort. Additionally, disease-associated transcriptional changes, used to derive a so-called molecular distance to health, were shown to correlate with radiographic changes and to revert to that of healthy controls after treatment. The difference in sensitivity between the UK and South African cohorts was attributed to the potential contribution of different Mtb lineages in the more ethnically diverse UK cohort as well as to latent TB cases being misclassified as active disease, potentially representing sub-clinical active TB.

A greater clinical challenge is distinguishing patients with TB disease from other diseases. In the study described above, Berry et al. [57] derived an 86-transcript signature of TB versus other inflammatory diseases, including staphylococcal and Group A streptococcal infections, systemic lupus erythematosus and Still’s disease, by comparison with previously published data sets. However, in subsequent studies, this did not discriminate TB from cases of pulmonary sarcoidosis [51], which can mimic the presentation of active TB. Bloom et al. [48] published a 144-transcript signature that could distinguish TB from other pulmonary disease (sarcoidosis, non-tuberculous pneumonia and lung cancer) and was derived from differentially expressed transcripts between the TB and sarcoid groups. When applied to training, test and validation cohorts, and using class prediction via support vector machines (SVMs), sensitivity was over 80 % with specificity over 90 % in distinguishing TB from non-TB (sarcoid, pneumonia, lung cancer, healthy controls). This study was restricted to UK and French patients and the number of patients with pneumonia or lung cancer was relatively modest. In addition, pneumonia cases compared to TB in this study experienced a variable duration of antibiotic therapy before transcriptomic sampling, which may have significantly confounded the conclusions as the authors highlighted the effect of antibiotic treatment on transcriptional profiles in a separate cohort of pneumonia patients.

Kaforou et al. [59] and Anderson et al. [60] presented much larger multi-centre studies in Africa, comparing TB to other diseases where TB was in the differential diagnosis. Importantly, these included HIV-positive adults and children, respectively, and encompassed a far broader range of conditions than the previously described studies [59, 60]. Kaforou et al. [59] recruited adult patients to compare culture-positive pulmonary and extrapulmonary TB to LTBI and other diseases. Discovery cohorts from Malawi and South Africa were used to define a 44-transcript signature of TB versus other diseases, which was then validated with an external dataset. They also proposed a calculation for a so-called Disease Risk Score (DRS) to reduce the multigene transcriptional signature to a single numerical value in order to discriminate TB from other diseases. This provided a sensitivity of 93–100 % in test and validation cohorts, and specificities of 88–96 %. The inclusion of a broad range of diagnoses represents a pragmatic approach relevant to clinical setting in which TB presents.

In their study of children with TB, Anderson et al. [60] employed a similar study design in the same geographical locations, although the description of TB disease was not detailed. The DRS based on a 51-transcript signature distinguished TB from other diseases in the validation cohort with a lower sensitivity of 82.9 % and specificity of 83.6 %. Additionally, culture-negative cases were included and evaluated separately. In this context, sensitivity decreased to as low as 35 % in the ‘possible TB’ cases but specificity was maintained at around 80 %. The DRS therefore outperformed the Xpert MTB/RIF assay in sensitivity in both culture-positive and -negative cohorts, but could not compete with the 100 % specificity of this PCR assay.

A new whole blood transcriptomic study in a US population identified new classifiers for active TB and compared their accuracy to those from other published studies [48, 51, 57, 59] using SVM and receiver operator characteristic curves [61]. They described high areas under the curve (AUCs) when discriminating between TB and pneumonia in their own cohort (0.965). These were higher than previously published signatures (0.9 and 0.82) and also performed well when used to classify a previously published dataset (0.906). In contrast, TB versus LTBI classifiers in all studies performed consistently accurately when applied across all datasets. Although not yet available at the time of writing, this study will provide additional array data valuable for cross validation in future studies. In this respect, an important hurdle to undertaking cross validation between published studies and meta-analyses is the lack of metadata linking individual cases to the corresponding transcriptomes deposited in public repositories.

Furthermore, the fact that all the diagnostic signatures described above are based on multigene signatures necessitates capacity for whole genome measurements or at least PCR multiplexing. The most recent study published by Maertzdorf et al. [62] aimed to identify the minimum number of transcripts that provide optimal diagnostic accuracy in order to reduce the cost of such tests and, therefore, their accessibility. Based on microarray datasets from two previous studies [50, 56], a 360-gene target custom made PCR array was applied to samples from a new cohort of TB patients and healthy controls in India. A stepwise approach using training and testing sets was used to define a small set of top classifying genes. Two tree-based models were used, with the conditional inference model identifying a four-gene signature (GBP1, ID3, P2RY14 and IFITM3) that could differentiate active TB from a healthy state with an AUC of 0.98. Independent validation using RT-PCR was performed in two further African cohorts resulting in AUCs of 0.82 and 0.89. The study goes on to analyse existing published microarray datasets, training their model on RT-PCR data of TB and healthy controls in India, and testing with microarray data from various studies [48, 51, 57, 59, 63]. The signature maintained consistently high AUCs in all HIV-negative populations when discriminating active TB from healthy states, but yielded a lower AUC in HIV-positive cohorts. Additionally, evaluation of its performance in ‘other disease’ cohorts was found to be more variable across varying ethnicities, geographical locations and HIV status. This is an exciting step forward identifying potential candidates for development in molecular point-of-care TB diagnostics.

#### Tissue transcriptomics

Blood samples are taken as part of routine clinical care, and thus are readily accessible for research purposes and diagnostic tests. However, transcriptional profiling at the site of disease may yield biologically relevant responses that are not evident in blood. Indeed, a blood signature that discriminates between individuals with active and latent TB infection is only partly enriched in the transcriptome of human TB lung granulomas [64] and cervical lymph nodes [65]. Similarly, the transcriptional signature that distinguishes TB from sarcoidosis in mediastinal lymph node samples shows little overlap with previously published peripheral blood signatures [66].

One explanation for these compartmentalised responses could be the structural heterogeneity that is observed amongst individual granulomas within the same host [67], and which is also reflected in the transcriptome [64]. Subbian et al. [64] found that fibrotic nodules showed both quantitatively and qualitatively different transcriptional changes compared to cavitating granulomas. The heterogeneity of localised tissue responses may be lost when averaging systemic (blood) responses, and potentially impede the discovery of sensitive peripheral blood biomarkers. Therefore, transcriptomes from the site of disease may provide more sensitive biomarkers than peripheral blood and complement conventional histopathological diagnostics [66].

#### Proteomics

Several studies have investigated the diagnostic potential of proteomic fingerprinting to identify different disease states (i.e. active TB versus healthy state, LTBI or other diseases) and monitor the treatment response in TB (Table 2).

**Table 2:** Proteomics studies

Study	Sample	Data access	Country	Classes	Number	Case definition						Independent test set^c^	Validation set^d^	Evaluation of accuracy	Signature size	Protein biomarkers identified
						HIV status	Prior TB treatment^a^	TB location	Microbiologically proven^b^	TST	IGRA					
Achkar et al., 2015 [77]	Serum	Y	US	TB	37	–	≤7d	P, EP	U			N	N	Y	10	Y
				LTBI	34	–				+	+/–					
				HC	20	–				–						
				OD	19	–										
				TB	10	+	≤7d	P, EP	U						8	
				LTBI	23	+				+	+/–					
				HC	16	+				–						
				OD	26	+										
Wang et al., 2015 [102]	Serum	N	China	TB	122	–	–	P	U			N	N	Y	5	Y
				Treated	91	–	2 m									
				Cured	59	–	≥6 m									
				HC	122											
Liu et al., 2015 [72]	Serum	N	China	SP-TB	49	–	–	P	Y			Y	N	Y	3	N
				SN-TB	66	–	–	P	Y							
				HC	80	–										
Xu et al., 2015 [69]	Serum	N	China	TB	40	–	–	P	Y			N	N	Y	3	N
				HC	40											
				OD	80											
Zhang et al., 2014 [103]	Plasma	N	China	LTBI	71	–				+	+	Y	N	Y	19	Y
				HC	75	–				–	–					
Xu et al., 2014 [92]	Serum	N	China	TB	76	–		P	U			N	N	Y	3	Y
				HC	56											
Song et al., 2014 [104]	Serum	N	South Korea	TB	26		–	P	U			N	N	Y	1	Y
				HC	31											
Nahid et al., 2014 [105]	Serum	N	Uganda	TB	39	–	≤5d	P	Y			N	N	Y	4	Y
				Responder	19	–	2 m									
				Non-responder	20	–	2 m									
Ou et al., 2013 [106]	CSF	N	China	EP-TB	45		–	EP	Y			N	N	N	N/A^e^	Y
				HC	45											
				OD	45											
Liu et al., 2013 [70]	Serum	N	China	TB	180	–	–	P	U			Y	N	Y	4	N
				HC	91	–										
				OD	120	–										
De Groote et al., 2013 [107]	Serum	N	Uganda	TB	39	–	≤5d	P	Y			N	N	N	N/A^e^	Y
				Treated	39	–	2 m									
Zhang et al., 2012 [71]	Serum	N	China	TB	129			P	Y	+		N	N	Y	3	N
				LTBI	36					+						
				HC	30											
				OD	69											
Sandhu et al., 2012 [73]	Plasma	N	Peru	TB	151			P	Y			N	N	Y		N
				OD (+LTBI)	53						+				33	
				OD (-LTBI)	44						–				57	
				OD all	110						+/–				98	
Liu et al., 2011 [75]	Serum	N	China	TB	80	–		P	U			Y	N	Y	3	N
				HC	32	–										
				OD	36	–										
Deng et al., 2011 [74]	Serum	N	China	TB	37	–	–	P	Y			Y	N	Y	5	N
				EP-TB	81	–	–	EP, P	U							
				HC	40	–					–					
				OD	35	–					–					
Tanaka et al., 2011 [108]	Plasma	N	Japan, Vietnam	TB	39	–	≤7d	P	Y			N	N	N	N/A^e^	Y
				HC	63											
Liu et al., 2010 [76]	Serum	N	China	SP-TB	51	–		P	Y			Y	N	Y	9	N
				SN-TB	36	–		P	Y						2	
				HC	55	–										
				OD	13	–										
Agranoff et al., 2006 [68]	Serum	N	Uganda, The Gambia, Angola, UK	TB	197	+/–	≤7d	P, EP	Y			Y	Y	Y	4	Y
				HC	25	+/–										
				OD	168	+/–										

*CSF* Cerebrospinal fluid, *TB* Active tuberculosis, *LTBI* Latent TB infection, *HC* Healthy controls, *OD* Other diseases, *SP* Smear positive, *SN* Smear negative, *EP* Extrapulmonary, *P* Pulmonary; *Y* yes, *N* no

^a^number of days (d) or months (m) on treatment at time of sampling

^b^U if unclear whether all TB cases were microbiologically confirmed, e.g. if diagnosis was based on Mtb culture or chest X-ray or TB symptoms, or if microbiologically proven and unproven TB cases were grouped together

^c^never involved in training the model (nested, k-fold or leave-one-out cross-validation (without test) are not considered to make use of an independent test set)

^d^new, independent set of samples, e.g. from different ethnic background or geographic location

^e^Differentially expressed proteins were identified but suitability as biomarkers was not assessed

In 2006, Agranoff et al. [68] were the first to demonstrate that the serum proteome can distinguish active pulmonary TB from both non-TB disease and healthy controls. Employing proteomic profiling and a SVM learning approach, this landmark study identified a combination of four biomarkers (serum amyloid A, transthyretin, neopterin and C reactive protein), that, when measured by conventional immunoassays such as ELISA, could identify active TB cases in an independent cohort with a sensitivity and specificity of 88 and 74 %, respectively. The authors hypothesised that diagnostic accuracy could be further improved with immunoassays that target specific protein variants (as identified by proteomic technologies) rather than the total protein.

Despite these early findings, and numerous studies since, there are at least two major barriers to translating proteomic biomarkers into diagnostic tests. Firstly, the protein biomarker candidates reported by independent studies vary considerably and a universal proteomic profile of TB has therefore remained elusive. Differences in proteomic techniques and their resolutions, study design, case definitions and statistical analyses may all contribute to discrepant results. Nevertheless, there is overlap in the proteins reported to be differentially expressed in active TB; selected examples include CD14, S100A proteins, apolipoproteins, fibrinogen, orosomucoid and serum amyloid A. The decision regarding which of these differentially expressed proteins are further considered or combined as candidate biomarkers can however be biased. For instance, investigators may choose to validate only proteins that can be measured by commercial ELISA kits [69], identify only (arbitrarily) selected differentially expressed protein peaks [70, 71] or none at all [72–76], or exclude ‘non-specific’ inflammatory markers such as acute phase proteins [77]. The inconsistent selection approach taken by different groups consequently impairs the assessment of common protein signatures between independent studies. Secondly, identified proteins of interest are not always (1) evaluated for their diagnostic potential (i.e. with receiver operator curve analyses or decision trees); (2) cross-validated in independent datasets; or (3) evaluated with external datasets.

Indeed, the need to validate diagnostic models in independently recruited patient populations and to define the target group in which the diagnostic test is likely to be successful (e.g. ethnic background, HIV status) has been convincingly illustrated by Ratzinger et al. [78], who applied the diagnostic algorithm previously devised by Agranoff et al. [68] to a new Central European patient cohort of 36 active TB cases and 170 patients with other diseases. The originally published diagnostic algorithm predicted disease status in the new cohort with a poor accuracy of only 54 % (19 % sensitivity and 62 % specificity). Ratzinger et al. [78] argued that the performance difference to the initial study may be attributable to differences in the composition of the comparison groups and the pre-test probability due to study design (approximately 1:1 distribution of TB cases and controls in the original case-control study [68] versus 1:4 distribution in the following cross-sectional study [78]).

Further, only one, very recent study has deposited its proteomic data on publically available databases. In this study, Achkar et al. [77] identified two separate protein biosignatures with excellent diagnostic accuracy for active TB in either HIV uninfected (AUC 0.96) or co-infected individuals (AUC 0.95). In this prospective study [77], the TB group included smear-negative and smear-positive as well as pulmonary and extrapulmonary cases. Despite the small number of patients, the resulting protein panels are likely to be useful in clinical practice if they can be cross-validated, and the deposited data represent a valuable external reference set for future studies.

Taken together, many studies have described alterations in peripheral blood proteins during active TB and suggested those as diagnostic disease markers. Although the observed differences evolve around common functional categories, in particular inflammatory responses, tissue repair and lipid metabolism [77], significant improvements in standardisation and validation procedures are needed to increase reproducibility and accuracy of protein biosignatures, and to advance adoption to the clinical setting [79].

#### Metabolomics

TB-associated changes in the metabolite profile have been examined in blood and other clinical specimens such as urine, sputum, cerebrospinal fluid or breath (Table 3). However, the primary aim of most published studies has been to gain novel biological insights into TB pathogenesis rather than to probe diagnostic value. Accordingly, diagnostic performance of the candidate biomarkers has not always been assessed. Those interested in a diagnostic evaluation cannot easily make use of the generated data since these are not routinely deposited on public databases, with only one study providing its raw data as supplementary material [80].

**Table 3:** Metabolomics studies

Study	Sample	Data access	Country	Classes	Number	Case definition						Independent test set^c^	Validation set^d^	Evaluation of accuracy^e^	Signature size	Metabolite biomarkers identified
						HIV status	Prior TB treatment^a^	TB location	Microbiologically proven^b^	TST	IGRA					
Zhou et al., 2015 [109]	Plasma	N	China	TB	?			P	Y			N	N	N	N/A^f^	Y
				HC	?					–	–					
				OD	110					–	–					
Lau et al., 2015 [80]	Plasma	Y	Hong Kong	TB	37		–	P	Y			N	N	Y	2	Y
				HC	30											
				OD	30											
Feng et al., 2015 [110]	Serum	N	China	TB	120			P	U			N	N	Y	4	Y
				HC	105											
				OD	146											
Mason et al., 2015 [111]	CSF	N	South Africa, The Netherlands	EP-TB	17	–		EP, P	Y			N	N	N	N/A^f^	Y
				OD	49	–										
Das et al., 2015 [82]	Urine	N	India	TB	21	–	–	P	Y			N	N	Y	42	Y
				OD	21	–	–									
Frediani et al., 2014 [86]	Plasma	N	Georgia	TB	17		≤7d	P	Y			N	N	N	N/A^f^	Y
				HC	17											
Mahapatra et al., 2014 [89]	Urine	N	Uganda, South Africa	TB	87	–	–	P	Y			N	N	Y	6	Y
				Treated	59	–	1 m									
				Treated	20	–	2 m									
				Treated	54	–	6 m									
Zhou et al., 2013 [112]	Serum	N	China	TB	38			P, EP	Y			N	N	N	N/A^f^	Y
				HC	39					–	–					
Che et al., 2013 [113]	Serum	N	China	TB	136	–	–	P, EP	U			Y	N	Y	1	Y
				Treated	6	–	2 m									
				HC	130	–										
Du Preez and Loots 2013 [87]	Sputum	N	South Africa	TB	34			P	Y			N	N	N	N/A^f^	Y
				OD	61											
Weiner et al., 2012 [81]	Serum	N	South Africa	TB	44	–	–	P	Y			N	N	Y	20	Y
				LTBI	46	–				+						
				HC	46	–				–						
Kolk et al., 2012 [85]	Breath	N	South Africa	TB	71		+	P	Y			Y	N	Y	7	Y
				OD	100											
Banday et al., 2011 [84]	Urine	N	India	TB	117		–	P	Y			Y	N	Y	5	Y
				Treated	20		≤7 m									
				LTBI	19					+						
				HC	37					–						
				OD	12											
Phillips et al., 2010 [114]	Breath	N	US, Philippines, UK	TB ^g^	226		–	P	U			N	N	Y	10	Y
Phillips et al., 2007 [83]	Breath	N	US	TB	23			P	Y	+/–		N	N	Y	130	Y
				LTBI	19					+						
				OD	59	+/–				+/–						

*CSF* Cerebrospinal fluid, *TB* Active tuberculosis, *LTBI* Latent TB infection, *HC* Healthy controls, *OD* Other diseases, *EP* Extrapulmonary, *P* Pulmonary, *Y* Yes, *N* No

^a^number of days (d) or months (m) on treatment at time of sampling

^b^U if unclear whether all TB cases were microbiologically confirmed, e.g. if diagnosis was based on Mtb culture or chest X-ray or TB symptoms, or if microbiologically proven and unproven TB cases were grouped together

^c^never involved in training the model (nested, k-fold or leave-one-out cross-validation (without test) are not considered to make use of an independent test set)

^d^new, independent set of samples, e.g. from different ethnic background or geographic location

^e^predictive ability of the (O)PLS-DA model was not considered a valid accuracy evaluation

^f^Differentially expressed metabolites were identified but suitability as biomarkers was not assessed

^g^Different diagnostic criteria were compared but class distribution was not clear

For the majority of studies that have evaluated the accuracy of metabolic biomarkers, it is unclear whether active TB cases were microbiologically proven since radiological disease was often included as a diagnostic criterion. This leaves only a few reports comparing confirmed active TB with healthy, LTBI or symptomatic disease controls. Weiner et al. [81] demonstrated that 20 serum metabolites sufficed to discriminate between patients with active pulmonary TB and healthy controls (with or without LTBI) with an accuracy of 97 %. Lau et al. [80] reported that the combination of the cholesterol precursor 4α-formyl-4β-methyl-5α-cholesta-8-en-3β-ol with either 12-hydroxyeicosatetraenoic acid or cholesterol sulphate differentiated active pulmonary TB not only from healthy controls but also from patients with community-acquired pneumonia with >70 % sensitivity and ≥90 % specificity. In urine, 42 compounds were needed to identify active TB cases amongst TB suspects with an AUC of 0.85 [82], while in breath, Mtb-derived volatile organic compounds predicted active TB patients amongst TB suspects with an AUC of 0.93 [83]. However, none of these studies included independent test sets. By contrast, Banday et al. [84] generated a model based on five urine metabolites (o-xylene, isopropyl acetate, 3-pentanol, dimethylstyrene and cymol) that, in an independent test set of active TB cases and healthy controls, achieved an AUC of 0.988. In addition, Kolk et al. [85] derived a seven-metabolite signature by breath analysis in a South African cohort of TB suspects, which in a different set of patients from the same area yielded 62 % sensitivity and 84 % specificity. It should be noted that a similar sensitivity (64 %) was achieved when the authors randomly assigned samples as TB or non-TB cases, whereas the specificity dropped to 60 %.

Alterations detected in the metabolome of active TB patients include differences in the abundance of specific host-derived metabolites but also the presence of compounds derived from Mtb itself (e.g. cell wall lipids) or – when including TB patients on treatment – of anti-TB drugs [86, 87]. It is therefore important to consider subject characteristics when comparing metabolite biosignatures reported by different studies. In addition, since the metabolic profile is shaped by several environmental factors, including dietary intake, medication, comorbidities and stress [88], careful matching of cases and controls is desirable during biomarker discovery to minimise metabolite ‘noise’. In the catalogued studies, only Frediani et al. [86] addressed this issue by assessing dietary intake and matching TB cases with healthy household controls.

The number of measured metabolites varies greatly between published studies (from 34 to >21,000), dependent on, for example, the analytical technique used. The difference in measured metabolites and the often large proportion of unidentifiable metabolite peaks render it difficult to compare biosignatures between studies or to reproduce findings. Indeed, Mahapatra et al. [89] had to exclude 10 of 45 potential biomarkers identified in the discovery set as they did not yield quantitative data in the test set despite consistent use of the analytical technique (liquid chromatography–mass spectrometry).

To summarise, the metabolomics approach to TB biomarker discovery faces many of the same challenges as proteomics, including data availability, reproducibility, standardisation and validation. The current lack of extensive cross-validation and of robust overlap between independent studies means that no satisfactory metabolite biosignatures have been discovered yet, and this emphasises the need for additional, well-designed studies aimed specifically towards the discovery of diagnostic markers.

## Conclusion

Current diagnostics are inadequate and -omics approaches provide evidence that it may be possible to use the host response to diagnose TB. However, there are common limitations to the -omics studies described and we suggest the following framework for future TB biomarker studies.

Firstly, TB case definitions (Box 2) and time of sampling need to be standardised and clearly distinguishable on a case-by-case basis. Since treatment effects on the transcriptome have been described as early as 1 or 2 weeks [54, 55], samples should ideally be taken pre-treatment.

Secondly, technical aspects of experiments, such as the mapping to registries, also require standardisation. For example, microarray cross-comparison problems arise when transcriptomic studies are performed using different platforms, and a move to RNA sequencing with standardised sequencing depth could bypass this problem. At the very least, biomarker discovery studies need to provide a clear and complete description of their methodologies to enable replication in follow-up studies with new cohorts, and therefore allow exclusion of experimental variability as a potential confounder.

Thirdly, ascertaining an adequate sample size to train classification algorithms is difficult and no consensus exists on a priori requirements. In the existing (transcriptomic) literature, sample size ranges from 3 to 883 patients. However, it is expected that, if an algorithm has been trained with an adequate sample size, then algorithm performance should not deteriorate when the training set sample size is further increased. Tomlinson et al. [66] have recently demonstrated one way of assessing this by using computational simulations to model increasing training set sample sizes, in which they showed that test accuracy improved as sample size increased.

Finally, further assessment of new biomarkers by cross-validation is an essential step in the evaluation of the signature. True cross-validation involves a test set that has never contributed to model training. For example, leave-one-out validation does not meet this criterion, whereas splitting a cohort to use one part exclusively for training and the other exclusively for testing does represent a valid approach. Open access to -omics data with well-annotated, case-by-case metadata would facilitate external cross-validation with truly independent test sets and, in addition, assist in evaluating the applicability of a signature in different contexts. Alternatively, multi-centre studies (e.g. including high and low transmission settings) would provide an ideal environment to define and validate a TB biosignature.

We expect that adherence to this framework would facilitate biomarker discovery. Ultimately, however, prospective clinical trials need to be designed to test the impact of a diagnostic biosignature on TB diagnosis and clinical outcomes.

In clinical practice, much of the diagnostic uncertainty in TB arises in cases which are smear-negative pending culture and where microbiological culture is more difficult, such as in extrapulmonary TB, which represents up to half of the TB seen in lower transmission settings like the UK [90]. Thus far, most of the reviewed studies have been performed in the context of pulmonary, usually smear-positive, TB. A large proportion of TB presents as pulmonary TB in high transmission settings, and it is reasonable, therefore, to initially describe the host response in this homogenous sub-group [1]. It would be useful to extend future studies to include evaluation in more challenging clinical situations, and to assess whether the proposed diagnostic biomarkers can predict the risk of reactivation or progression of LTBI to active TB. In fact, the often moderate sensitivity and specificity achieved by diagnostic models based on -omics measurements may be of particular relevance for the unmet diagnostic need of such challenging settings. The World Health Organization has suggested optimal biomarker test requirements to detect TB as providing sensitivity ≥80 % in microbiologically confirmed extrapulmonary TB and ≥68 % in smear-negative culture-positive pulmonary TB [91]. Such requirements are met by some of the proteomic studies that distinguished extrapulmonary TB from other cases (including pulmonary TB, healthy controls and other disease) with a sensitivity of 94.4 % [74], and smear-negative TB from healthy controls with a sensitivity of >80 % [72, 76].

Alternatively, it may be more suitable to use -omics-based tests as triage tests to rule out TB when a high sensitivity can be reached but with lower specificity. The suggested minimum requirements for a TB triage test have been set out as >90 % sensitivity and >70 % specificity [91]. Again, these requirements have been met by some of the published studies [57, 59–61, 69, 81, 92]. However, substantial technical progress is needed to reduce price, equipment requirements and time for sample analysis and thus to make -omics tests adequate for field use [91].

Finally, while signatures containing multiple biomarkers (proteins, metabolites or transcripts) are more likely to be successful in identifying active TB, it is still worth exploring strategies that can reduce these to facilitate translation into diagnostic tests. For example, a minimal set of genes with a high diagnostic accuracy could be measured by more conventional techniques (e.g. PCR) in the field as demonstrated by Maertzdorf et al. [62]. It is unlikely, however, that one signature will be adequate to diagnose active TB in all clinical settings and it is more conceivable that different combinations of biomarkers will confer diagnostic value in different settings, e.g. one set of markers for differentiating between active and latent TB, and another to diagnose TB in comparison to other diseases.

## Box 1 High-throughput technologies to profile the host response in TB [93]

### Transcriptomics

Transcriptomics is the analysis of genome-wide gene expression, measured as RNA transcript abundance by gene chip microarrays or RNA sequencing. Most often, transcriptomics studies focus on the expression of protein-coding genes. However, the human transcriptome also includes non-coding RNA, and may contain up to 350,000 different transcripts [94]. Gene expression data from published transcriptomics studies are generally deposited in the public data repositories Gene Expression Omnibus (http://www.ncbi.nlm.nih.gov/geo/) or Array Express (https://www.ebi.ac.uk/arrayexpress/). However, the lack of detailed metadata and the use of different platforms render it difficult to combine individual datasets [95].

### Proteomics

Proteomics is the study of the collective set of proteins expressed by a cell or an organism at any given time. The human proteome is estimated to encompass up to one million different proteins. The main technology applied in proteomic studies is mass spectrometry, which involves fragmentation of proteins prior to their detection and quantification based on the mass-to-charge ratio of the resulting peptides. The detected peaks are first identified as peptides through a database search, and are then assigned to proteins through the use of identification algorithms [96].

### Metabolomics

Metabolomics aims to characterize the small molecule metabolites (e.g. lipids, fatty acids, sugars, amino acids, nucleotides) present in a clinical specimen. Approximately 20,000 different metabolites have been detected in human samples [97], with mass spectrometry and nuclear magnetic resonance as main detection tools. Examples of the analytical challenges associated with metabolomics studies include the dependency of the metabolite profile on the experimental methodology employed, and the broad spectrum of metabolite origin (e.g. drugs, nutrition) which need to be taken into account when interpreting inter-individual differences.

## Box 2 Standardised case definitions for TB based on World Health Organization criteria [1]

### Active disease

1. Bacteriologically confirmed TB
2. Presumptively treated TB

### Latent infection

The presence of immune responses to Mtb antigens (IGRA or TST positive) without clinical evidence of active TB
